# Study on PM2.5 pollution and the mortality due to lung cancer in China based on geographic weighted regression model

**DOI:** 10.1186/s12889-018-5844-4

**Published:** 2018-07-27

**Authors:** Qilong Cao, Guoqiang Rui, Ying Liang

**Affiliations:** 1 Business School, Changzhou University, Changzhou, Jiangsu Province People’s Republic of China; 2 Soochow University and Collaborative Innovation Center for New-type Urbanization and Social Governance of Jiangsu Province, Suzhou, Jiangsu People’s Republic of China; 3 Department of Social Work and Social Policy, School of Social and Behavioral Sciences, Nanjing University, 163 Xianlin Avenue, Qixia District, Nanjing, 210023 People’s Republic of China

**Keywords:** Geographic weighted regression, PM2.5, Lung cancer mortality, China

## Abstract

**Background:**

PM2.5 has become a major component of air pollution in China and has led to a series of health problems. The mortality rate caused by lung cancer has reached the point where it cannot be ignored in China. Air pollution is becoming more and more serious in China, which is increasingly affecting people’s lives and health.

**Methods:**

Considering the variations in the geographical environment in China, this paper studied the relationship between PM2.5 concentration and lung cancer mortality based on the geographical weighted regression model in 31 provinces in 2004 and 2008, autonomous regions and municipalities of China.

**Results:**

The results indicated there was a significant positive correlation between PM2.5 concentration and lung cancer mortality (*r* = 0.0052, *P* = 0.036). Additionally, the longer the time of exposure to PM2.5 is, the higher morbidity is.

**Conclusion:**

It is suggested that the Chinese government should launch some environmental policy, especially in those areas with severe PM2.5 pollutions, and keep the citizens away from exposure to PM2.5 pollution in the long term.

## Background

Lung cancer is also known as primary bronchogenic lung cancer. It is a malignant neoplasm with the fastest mortality. Which could affect human life seriously [1]. The latest data from the International Agency for Research on Cancer (IARC) shows that, the mortality rate for lung cancer is at the top of all malignancies worldwide. Its morbidity ranks first of all malignancies in male and ranks third in females, and showing an alarming increasing trend [2]. The phenomenon also occurs in China [3]. According to the China National Health and Family Planning Commission (CNHFPC), lung cancer ranks first in the mortality of malignant tumors. In 2015, the mortality rate of lung cancer in urban residents was 49.40/10 million, and the number of rural residents was 42.72/10 million [4].

The persistent growth in lung cancer’s morbidity and mortality has attracted the attention of many researchers. Studies have shown that many factors can cause lung cancer, such as smoking behavior [5], genetics [6], diet habits [7] and so on. Recently, as environmental issues intensify globally, many researchers have found that air pollution is also one of the important causes of morbidity and mortality growth of lung cancer [8]. For example, Chen et al. found that long-term exposure to air pollution will increase the possibility of people suffering from lung cancer [9]. Hamra et al., [10] showed that there is a significant correlation between PM2.5 and lung cancer. Therefore, we should pay great consideration to the relationship between air pollution and lung cancer.

At present, air pollution has become a global problem that threatens human survival and sustainable development. Air pollutants include ozone, particulate matter, nitrogen dioxide and sulfur dioxide, etc. The World Health Organization (WHO) has made clear regulations for the maximum limits of these air pollutants for the concentration of safety [11]. At present, PM2.5 are beyond the standard restrictions in many countries, especially in China. With the increase in haze, the harm of PM2.5 is gradually expanding, and has become the most threatening air pollutants [12, 13]. In 2013, China’s Ministry of Environment carried out PM2.5 monitoring work in 74 cities. The PM2.5 annual average concentration is between 26 and 160 μg / m3, and the average value is 72 μg / m3, which is the 2.06 times the annual average second level standard (35 μg / m3) by the environment air quality standards in China [14]. PM2.5 is caused by a variety of factors, such as traffic emissions, as well as dust and coal [15]. Considering the severity of Pm2.5 pollution in China, it is necessary to investigate its impact on the citizens’ health.

Epidemiological studies have shown that PM2.5 will pose serious threats and harm to human health. It is shown that PM2.5 can harm the respiratory system, the cardiovascular system [16, 17], the nervous system [18] and the immune system [19] and so on. It can further influence the national health [20], economic and social development [21] in the long term seriously. Researchers indicated that about 400,000 premature children died of PM2.5 each year in EU countries, especially for Poland where they suffered from the largest PM2.5 concentration [22]. Another study showed that as the daily average concentration of PM2.5 increased by every 10 μg / m3, the admission rate of coronary heart disease increased by 1.89%, the admission rate of heart attack increased by 2.25%, morbidity of congenital coronary heart disease increased by 1.85%, and respiratory disease risk increased by 2.07% [23]. And every 3.9 μg / mL reduction in PM concentrations will result in a reduction of 7978 hospitalizations and savings of approximately 333 million dollars. China’s economy loses 6.9 and 5.9% of GDP in 2000 and 2005 due to air pollution [24].

PM2.5, which is also known as fine particles, refers to particles with an aerodynamic equivalent diameter less than or equal to 2.5 μm in ambient air. It can be suspended in the air for a longer period of time. The higher its concentration in the air, the more severe the air pollution is. Compared with coarser airborne particles, PM2.5 has a small particle size, so it can go through the bronchus to affect the gas exchange in the lungs, resulting in the body’s hypoxia, and thus exerting a great influence on human health. Therefore, PM2.5 is an important risk factor for lung cancer [25, 26]. A 26-year cohort study for 188,699 non-smokers in Canada found that PM2.5 was positively correlated with lung cancer mortality [27]. Forastiere et al. [28] found that when PM2.5 increased by 10 μg / m3 it will result in lung cancer mortality increasing by 0.75% among the elderly living in Italy. The relationship between PM2.5 and lung cancer is also applicable to China. For example, studies found that the attribution factor of PM2.5 on lung cancer was 37.1, 35.9 and 34.9%, 3.7 ‰ in 2007–2009 respectively [29].

So far researchers have generally determined the positive relationship between PM2.5 and lung cancer. Still there are some questions that remain unsolved. As far as we are concerned, we found that there were relatively few prospective studies for the relationship between PM2.5 and lung cancer. Some were cohort studies but with inadequate PM2.5 sources or based on the short term [30]. And some used the cross-sectional data or panel data for analysis, which cannot reflect the dynamic characteristics of PM2.5 and spatial correlation characteristics.

This paper particularly focusses on the impact of PM2.5 on lung cancer for two reasons. Firstly, the mortality rate caused by lung cancer has reached the point where it cannot be ignored in China. Studies have shown that the morbidity and mortality of lung cancer in China has become the greatest threat to the Chinese people’s health and is showing a growing trend [14]. Secondly, air pollution is becoming more and more serious in China, which is increasingly affecting people’s lives and health [31]. Therefore, the aims of this study were to examine the relationship between PM2.5 concentration and lung cancer mortality in 31 provinces of China. Compared with the existing research, the main contributions of this paper are: (1) Based on the spatial perspective, the regional distribution of PM2.5 in China during 2004–2008 was analyzed, and the concentration distribution characteristics of PM2.5 in different administrative divisions would be revealed; (2) Considering the spatial heterogeneity of the variables used in this paper, this paper extended the traditional linear regression model, and constructed a geographic weighted regression model based on spatial relations, in order to reflect the spatial correlation between PM2.5 and lung cancer mortality; (3) This paper also compared and analyzed estimation results in different years, studied the time-dependent changes characteristics of the correlation between PM2.5 concentration and lung cancer mortality, in order to reflect the dynamic characteristics of the relationship between PM2.5 and lung cancer mortality.

## Methods

### Variable and data source

The dependent variable was lung cancer mortality and the independent variable was PM2.5 concentration. Studies have also shown that regional economic development [32], population density [33] and per capita medical costs [34] can also affect public health.^1^ Taking into account the influence of these factors, this paper selects the per capita GDP (Gross Domestic Product) of each province, the number of general hospitals in each province, the population density of each province and the per capita medical expenses of each province as the proxy variables of the above factors. In addition, in order to avoid the influence of heteroscedasticity and the difference between variable units, this paper deals with the natural logarithm for variables with high magnitude.

For data selection, the dependent variable (lung cancer mortality) came from the “China Disease Detection System Death Monitoring Network Report Database”. Due to the access constraints of the database, this paper can only get the relevant data from 2004 to 2008. Therefore, we chose lung cancer death number per 10,000 in from 2004 to 2008 as Proxy indicator of lung cancer mortality in 31 provinces, autonomous regions and municipalities (data lacked that of Taiwan, Hong Kong, Macao). The PM2.5 concentration (ug / m3) referred to the research results of Van Donkelaar et al. [35] and extracted the PM2.5 concentration value from 2004 to 2008 as a sample. The economic development level (lngdp) and population density (popu) were taken as control variables, retrieved from the China Statistical Yearbook. Another two independent variables, the per capita medical expenses (lncost) and the number of provincial general hospitals (lnmedi), are derived from the China Health Statistics Yearbook.

### Analysis model

In traditional regression, such as panel data analysis, it is generally assumed that the modeling relationship holds everywhere in the study area, namely, the regression parameters are “whole-map” statistics. In many situations, such as this study, this is not the case. As a developing country, there are great differences among provinces in China, especially the economic development among regions is uneven, and the gap among rich and poor province is very large. The developed provinces are almost concentrated in the southeastern coastal areas of China, such as Beijing, Shanghai, Jiangsu, and Zhejiang, while the poorer provinces in China are concentrated in the northwest and southwest regions, such as Guangxi, Yunnan, and Guizhou province, Qinghai Province, Gansu Province, etc. In addition, developed regions have more stringent environmental protection systems, while in less developed regions, environmental protection measures are very weak, such as the western provinces. Apparently, the panel model does not take the spatial heterogeneity of variables into account.

As a popular method derived from spatial econometric for analysis of spatial data, spatial weighted regression model is different from panel data analysis. In spatial weighted regression model, spatial data was used. This data has spatial utility, namely, spatial correlation and spatial heterogeneity. In addition, the other advantages of the GWR model is that it can use the sub-sample information of the adjacent region to perform local regression. It can estimate the parameters variables in each region, and then use the spatial visualization method to conduct spatial comparative analysis of different geographical regions. Although the Geographically Weighted Regression (GWR), one of spatial varying coefficient regression model, is an extension of the general linear regression model, it can reflect the spatial heterogeneity of variable distribution. Therefore, it is an effective method to solve the problem of spatial instability. Its formula is:

$$ {y}_i={\beta}_0\left({u}_{i,}{v}_i\right)+\sum \limits_{j=1}^k{\beta}_k\left({u}_{i,}{v}_i\right){x}_{k,i}+{\varepsilon}_i $$

Among them, (*u*_*i*,_*v*_*i*_) is the i-th the spatial coordinates (either geographical coordinates or projection coordinates). In this paper, we used the coordinates of the provinces to represent the spatial coordinates of each region. *y*_*i*_ is lung_death, the dependent variable in this paper. *x*_*k*, *i*_ are independent variables and control variables, as described above. In the GWR model, the regression parameters of a particular location no longer used all observations to carry out global estimates, but using sub-sample information for the adjacent area to carry out local regression estimates. The parameters of the estimated variables will be adjusted as the spatial position changes. In the GWR model, the estimated values of the parameters, after locally weighting neighborhood locations, can be expressed as:

$$ {\beta}_k\left({u}_i,{v}_i\right)={\left({X}^TW\left({u}_i,{v}_i\right)X\right)}^{-1}{X}^TW\left({u}_i,{v}_i\right)Y $$

Among them, *X* and *Y* are the independent variable matrix and the dependent variable matrix, respectively. *W*(*u*_*i*_, *v*_*i*_) is the weight matrix of the geometric weighted regression at the spatial position i. According to Brunsdon et al. (1996), this paper chose the Gaussian distance function as the basic form of spatial weight. That is,

$$ W\left({u}_i,{v}_i\right)=\varnothing \left({d}_{ij}/\sigma \theta \right) $$

Among them, *d*_*ij*_ is the geographical distance between the i-th spatial position and the j-th spatial position. ∅ is the standard normal distribution density function. *σ* is the standard deviation of the distance vector. *θ* is the bind width of the vector. This paper used the least squares method to estimate the parameters in the GWR model.

## Results

### Spatial distribution of PM2.5

**Fig. 1:**
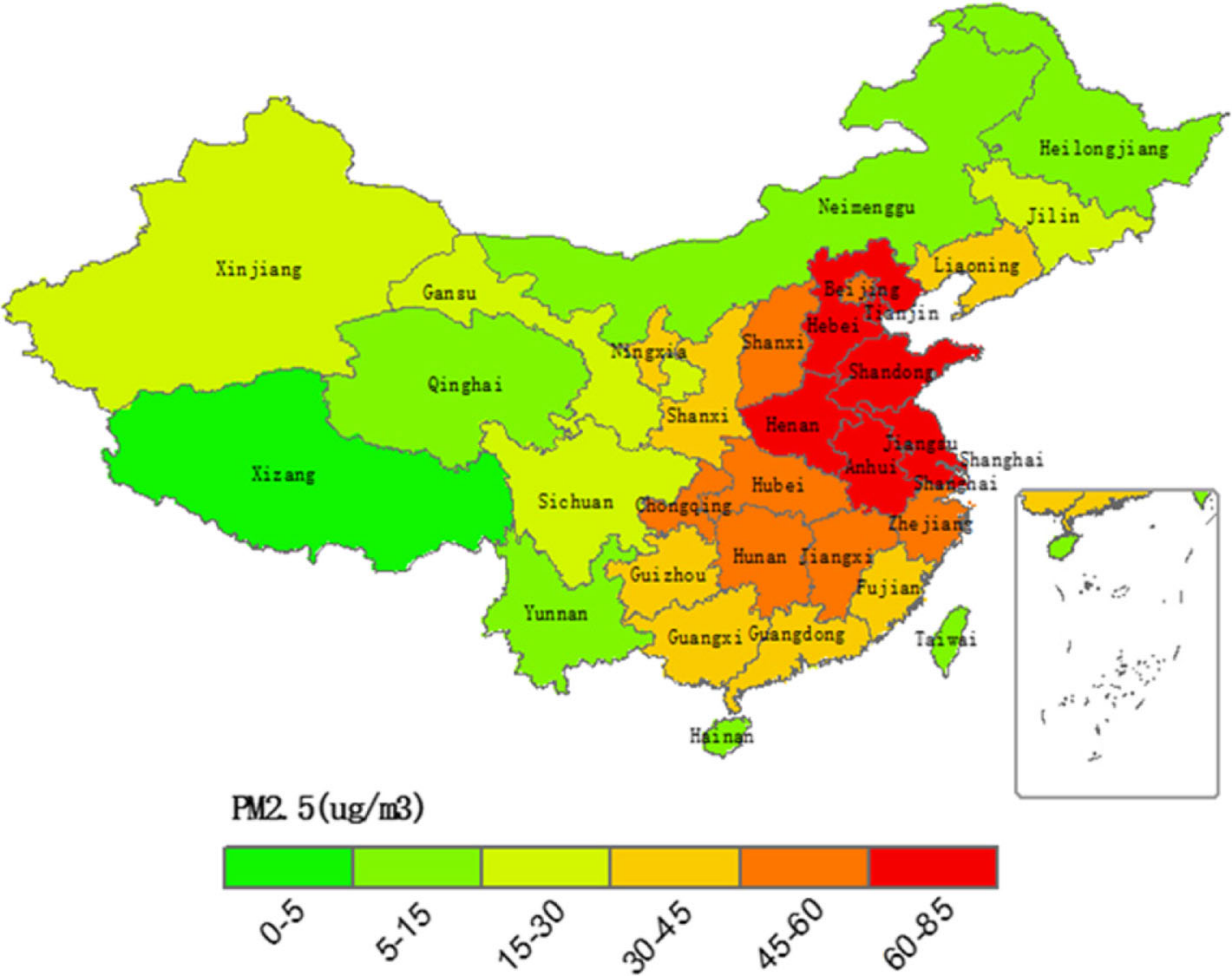
Average PM2.5 concentration for each province in China from 2004 to 2008. The map was drawn by the authors with ArcGIS software (a geographic information system for working with maps and geographic information) based the PM2.5 concentration data extracted at http://sedac.ciesin.columbia.edu/data/sets/browse

Figure 1 shows the five-year average PM2.5 concentration calculated for each province in China from 2004 to 2008. It can be seen from Fig. 1 that PM2.5 concentrations in most regions of China are relatively high, with 21 provinces accounting for more than 30 μg / m3 of PM2.5, accounting for 66% of the total number of administrative regions. In addition, it can be seen that the spatial agglomeration of PM2.5 pollution in China is obvious. The areas with the most serious pollution (PM2.5 concentration of 60-85μg / m3) are concentrated in the eastern areas of China, such as Tianjin, Hebei, Shandong, Henan, Jiangsu, Anhui and Shanghai. The average PM 2.5 concentration in Beijing, the capital of China, is 45–60 μg / m3, lower than the neighboring provinces and cities. This may stem from the 2008 Olympic Games held in Beijing. The Chinese government has adopted a series of policies to control air pollution.

### Lung cancer mortality and PM2.5 scatter plot

**Fig. 2:**
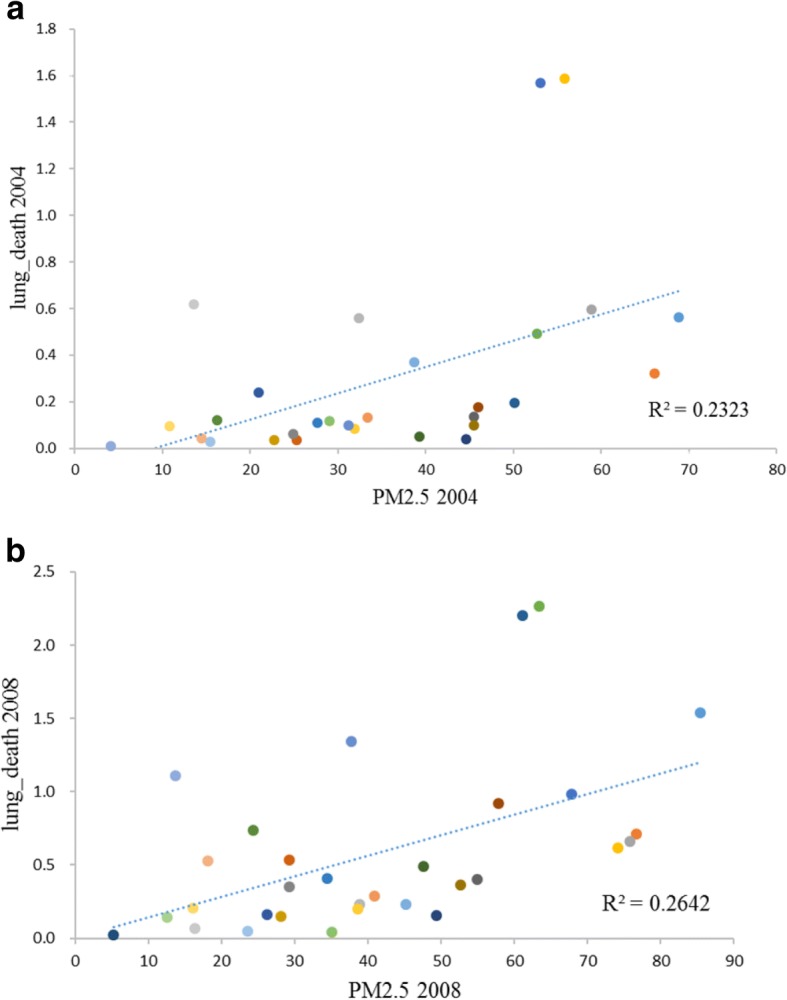
PM2.5 concentrations and lung cancer mortality scatter grams for 2004 (**a**) and 2008 (**b**) in 31 provinces in China

Figure 2 presented the data of lung cancer mortality and PM2.5 concentration in 2004 and 2008 in 31 provinces, autonomous regions and municipalities of China. The horizontal axis is the PM2.5 concentration value, the vertical axis is the lung cancer mortality rate and the dotted line in the figure is the trend fitting line. Fig. 2a is the scatter spot of the lung cancer mortality rate and the PM2.5 concentration value in 2004, and the Fig. 2b is a scatter plot of those in 2008. It can be seen that the lung cancer mortality and PM2.5 concentrations in 2004 and 2008 showed a linear relationship. Estimation results indicated R^2^ in Fig. 2a was 0.2323, indicating that PM2.5 in 2004 could explain 23.23% of the lung cancer mortality. And R^2^ in Fig. 2b was 0.2642, indicating that PM2.5 in 2008 could explain 26.42% of lung cancer mortality. The explanatory power of PM2.5 on lung cancer mortality in 5 years (2004–2008) increased by 13.73%. In addition, China’s PM2.5 was 41.36μg / m^3^ and lung cancer mortality rates were 0.53 / million in 2008, respectively, which were higher than those of PM2.5 (37.48μg / m^3^ in 2004) and lung cancer mortality (0.27 / million in 2004) in China in 2004.^2^ Therefore, compared to Fig. 2a, the coordinates of the points in Fig. 2b correspond to higher concentrations of PM2.5 and a higher lung cancer deaths rate, indicating China’s PM2.5 and lung cancer mortality rates are showing an increasing trend.

### GWR estimation results

GWR model is the expansion of the traditional linear regression model, which means we should add spatial geographic coordinates into the original model. In general, the first step of GWR models requires traditional linear regression, in order to validate the rationality to select index from the extended GWR model.

The results of the linear regression based on the least square method are shown in Table 1. The R2 and adjusted R2 indicate the model fitting is better. And the estimated coefficients of PM2.5 indicated lung cancer mortality and PM2.5 is significantly and positively related (B = 0.0052, *P* < 0.05). Some other control variables were also significant. For example, lngdp variable was significant at the 10% level, lncost, lnmedi and popu were significant at the 1% level. Therefore, it could be suggested that variables chosen in this paper are reasonable, we could continue to perform geo-weighted regression.

**Table 1:** Linear regression model estimation results

	Coef.	Std. Err	T	P
PM2.5	0.0052	0.0024	2.09	0.036
lngdp	0.2055	0.1063	1.93	0.055
lncost	1.0006	0.1799	5.56	0.000
lnmedi	0.1141	0.1263	0.90	0.366
popu	0.0004	0.0001	4.07	0.000
intercept	−4.9654	0.9515	−5.22	0.000
R^2^ = 0.6461, Adj R^2^ = 0.6317.				

lngdp: per capita gross domestic product; lncost: per capita medical expenses; lnmedi: number of provincial general hospitals; popu: population density; Coef.: estimated coefficient; Std. Err: standard error; T: T-Values; P: *p* values

**Fig. 3:**
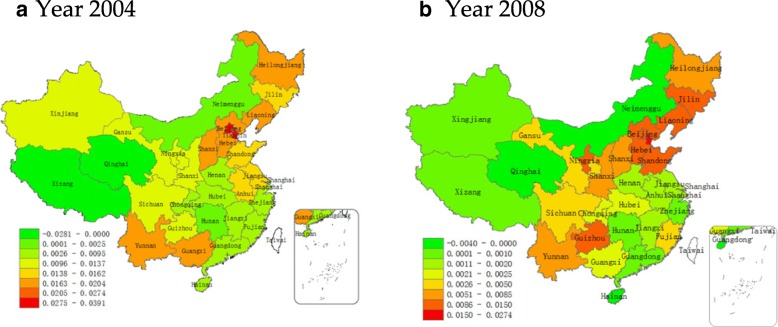
Spatial distribution of estimated coefficients of PM2.5 in (**a**) 2004 and (**b**) 2008 in various provinces, autonomous regions and municipalities of China. The map was drawn by the authors with ArcGIS software (a geographic information system for working with maps and geographic information) based the results obtained from the geographic weighted regression analyses

Table 2 presents the results of the GWR model for 2004 and 2008. This paper used the cross-sectional data, so it adopted a comparative analysis. Geographically weighted regression could report estimated coefficients for each variable in each province. Therefore, there will be 31 estimated coefficients for each variable, corresponding to 31 provinces. In order to compare them between different period, the average, median, minimum, and maximum values of the 31 estimated coefficients were calculated and reported in Table 2. Generally, the mean value of PM2.5 estimated coefficients was 0.0047 in 2004 and 0.0096 in 2008. It is worth noting that in the minimum value of PM 2.5 are negative in 2004 and 2008 (the negative regression coefficient will be reported in Fig. 3). It is assumed that some provinces had small coverage of monitoring, or few monitoring data and low PM2.5 concentration. For other variables, a similar pattern is shown in the results from Table 2, that is, overall, in addition to the variable popu, other variables (i.e. lngdp, lncost and lnmedi) have bigger estimated coefficient values in 2008 than that in 2004. In addition, the minimum value for all estimated coefficient are negative, which is also possible due to the fact that some provinces have less monitoring data.

**Table 2:** GWR model estimation Results

	Mean		Median		Min		Max	
	Year 2004	Year 2008	Year 2004	Year 2008	Year 2004	Year 2008	Year 2004	Year 2008
PM2.5	0.0047	0.0096	0.0019	0.0110	−0.0040	− 0.0281	0.0274	0.0391
lngdp	0.1032	0.1559	0.1399	0.1823	−1.0014	−1.1763	0.5566	0.6384
lncost	0.4422	1.2551	0.1921	0.6807	−1.3661	−0.5790	2.0427	6.5961
lnmedi	−0.1449	0.2783	−0.1103	0.1668	−0.8896	−0.6085	0.1797	1.9552
popu	0.0002	−0.0001	−0.0011	0.0009	−0.0052	− 0.0086	0.0018	0.0032
intercept	−2.0213	−1.3147	−0.4548	−5.6989	− 21.0838	−7.9364	4.7124	5.2347
R^2^	0.5660	0.6774	0.5660	0.6774	0.5660	0.6774	0.5660	0.6774
Adj R^2^	0.4395	0.5833	0.4395	0.5833	0.4395	0.5833	0.4395	0.5833

lngdp: per capita gross domestic product; lncost: per capita medical expenses; lnmedi: number of provincial general hospitals; popu: population density

Geographically weighted regression could report estimated coefficients for each variable in each province. Therefore, there will be 31 estimated coefficients for each variable, corresponding to 31 provinces. In order to compare them between different period, the average, median, minimum, and maximum values of the 31 estimated coefficients were calculated and reported in Table 2

One of the advantages of the GWR model is that it can use the sub-sample information of the adjacent region to perform local regression. It can estimate the parameters variables in each region, and then use the spatial visualization method to conduct spatial comparative analysis of different geographical regions. Figure 3 showed the spatial distribution of the estimated coefficients of PM2.5 in 2004 and 2008 in 31 provinces. The subgraph (a) showed the coefficients in 2004, and subgraph (b) showed in 2008. It can be seen that the estimated coefficients of PM2.5 showed certain characteristics in space. The estimated coefficient gradually increased from west to east. Also there were obvious differences between the different regions. The PM2.5 high coefficients gathered spatially, especially in Beijing, Tianjin, Hebei and other regions where they have a higher PM2.5 coefficient estimates. As can be also seen from Fig. 2, these regions are also the most polluted areas in China. In contrast PM2.5 coefficients are also smaller in areas where PM2.5 is less polluted. From the map, this relationship does not show a strict one-to-one relationship. For the more stringent consideration, this paper tested the correlation coefficient of PM2.5 concentration and the regression coefficient of PM2.5 on lung cancer in linear regression. The correlation coefficients for 2004 and 2008 were 0.76 and 0.87, respectively, and were significantly positively correlated at 5 and 1%, respectively. Therefore, it can be concluded that there is a positive correlation between PM2.5 concentration and lung cancer mortality, and the intensity of this relationship varies with the geographic location associated with PM2.5 concentration.

### PM2.5 estimating the dynamics of the coefficient time span

**Fig. 4:**
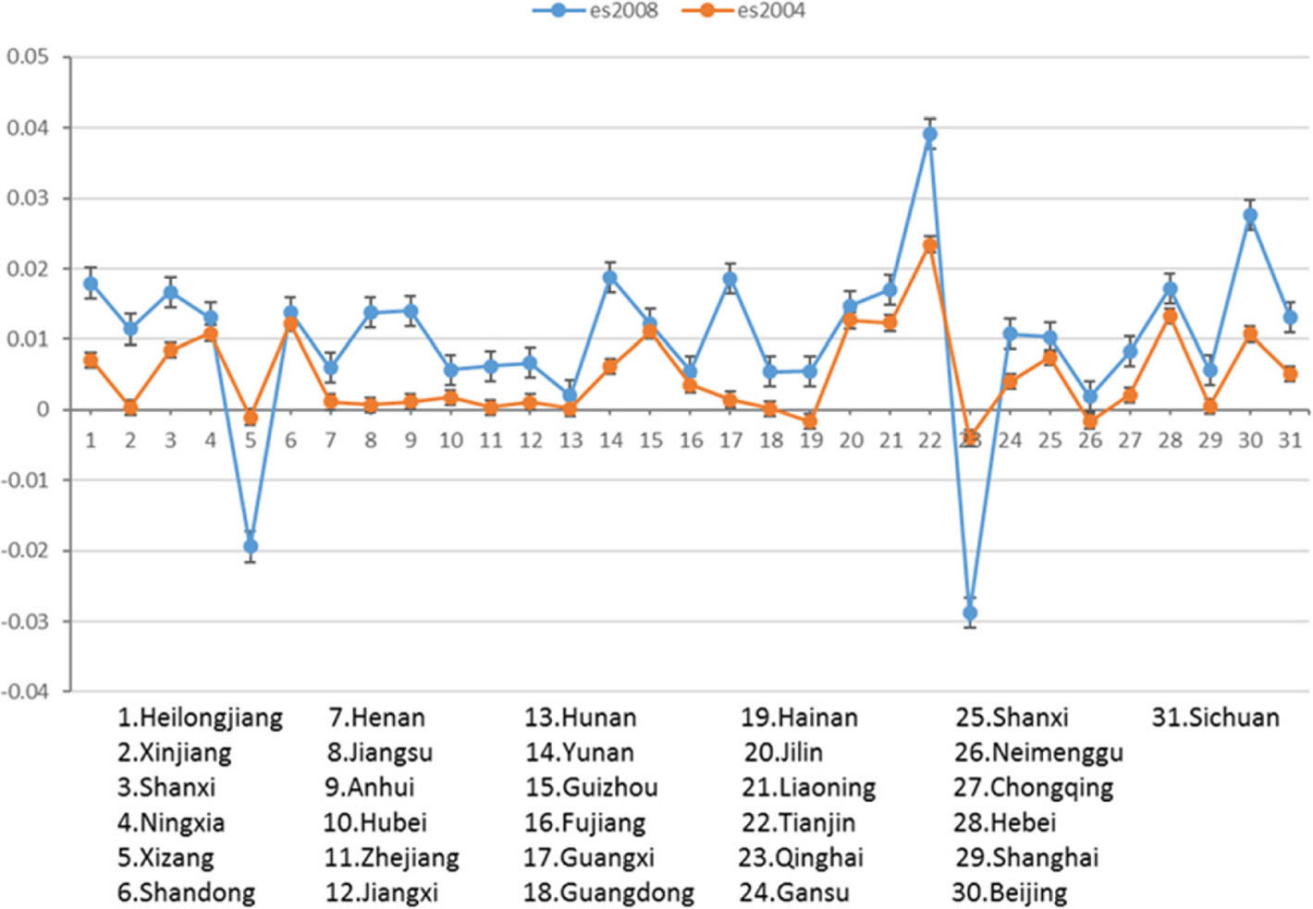
Comparison of PM2.5 estimated values for geographic weighting regressions in 2004 and 2008 in provinces, autonomous regions and municipalities in China

Previous studies have shown that the mortality is not only related to the concentration of PM2.5, but also the time exposed to PM2.5. That is, more exposure to a high concentration of PM2.5 environment, the higher the mortality is. In order to verify the applicability of this conclusion in China, the trend of regression coefficient of PM2.5 in 2004 and 2008 is analyzed in this paper. Since the same province in 2008 suffered longer from PM2.5 than in 2004, so the change in the coefficient in different years represents the intensity of PM2.5 on lung cancer mortality changes. Figure 4 shows the correspondence between PM2.5 estimated coefficients in different provinces in the form of a line graph. The horizontal axis 1 to 31 represents the 31 provinces in China. The vertical axis is the estimated coefficient of the GWR model. It can be seen from Fig. 4 that, in general, the fluctuation range of the estimated coefficient of PM2.5 in 2008 is larger than the fluctuation range of the estimated coefficient of 2004. Excluding provinces with negative estimated value (Qinghai province and Tibet autonomous region), the estimated coefficient in 2008 is higher than that in 2004, which indicates that the intensity of PM2.5 on lung cancer mortality has improved from 2004 to 2008 to a certain extent. Therefore, we can infer that the longer the exposure to PM2.5, the greater the impact of PM2.5 concentration on lung cancer mortality.

## Discussion

This paper used large scale data from 31 provinces, autonomous regions and municipalities in China. Also it used the GWR model and spatial visualization technique. We used the longitudinal and horizontal comparisons to study the relationship between PM2.5 concentration and the lung cancer mortality. Two main findings emerged from this paper.

Firstly, there is a significant positive correlation between PM2.5 concentration and lung cancer mortality. According the results of linear regression in Table 1, at the 5% significance level, PM2.5 concentration value increases by 10μg / m3, the lung cancer mortality increased by 5.2% correspondingly. In addition, the correlation between the concentration of PM2.5 and the mortality of lung cancer showed a certain spatial distribution with the difference of PM2.5 concentration. Namely, the higher the PM2.5 concentration in some regions is, the greater the intensity of the correlation with lung cancer mortality, and vice versa.

Secondly, with the time going by, the intensity of the impact on lung cancer mortality rate will increase. By comparing the estimates of the coefficients of PM2.5 in 2004 and 2008 in Chinese provinces, autonomous regions and municipalities, this paper holds that the estimate of PM2.5 in 2008 is higher than the estimate of the coefficient in 2004 overall. From 2004 to 2008, the effects of PM2.5 on lung cancer mortality rate has shown a certain degree of improvement.

In addition, for the control variables, the impact of regional economic development level in Table 1 is significantly positive at 10% level. It could be explained as the economic development model being pursued at the expense of the environment pollution in China. Figure 1 also illustrated that the areas with high PM2.5 concentrations are distributed in the eastern China, such as Tianjin, Beijing, Shandong, Jiangsu and other places. The higher PM2.5 concentrations also clearly presents a high level of lung cancer mortality, resulting in the positive correlation between the level of economic development and lung cancer mortality rate. Similarly, lnpopu, the population density index is significant at the 1% level, and the area which is the most densely populated in China, is precisely the region with higher concentration of PM2.5. The significant positive correlation between per capita medical expenses and lung cancer mortality at 1% level in Table 1 is also a scenario that is not consistent with normal expectations. This paper suggests that it may be the indicator itself, and due to the fact that per capita medical costs include the average cost of all treatment, the lung cancer only attributes a small part. So this paper holds that this indicator only reflects the current rapid increase in China’s annual medical expenses and lung cancer mortality. While the other variable, lnmedi, the quantity indicator of regional hospitals presents negative correlation with lung cancer mortality. This indicates that, to a certain extent, a better availability of medical services inhibits lung cancer mortality.

It should be noted that there is a significant positive correlation between PM2.5 and lung cancer mortality as illustrated in Table 1. But in the GWR model, some regression coefficients of PM2.5 are negative (Tibet, Hainan, Qinghai and Inner Mongolia in 2004; Tibet and Qinghai in 2008). This paper argues that the main reason for this is the difference between the model itself and the availability of data. Because the data from all provinces, municipalities and autonomous regions in China are integrated by the linear regression model, the GWR model only uses regional data to regress. Also as the sampling points of lung cancer mortality in China are mostly from economically developed areas and less so in remote areas, therefore this paper suggests that the monitoring data in these areas cannot fully reflect the real relationship.

Based on these findings, the contribution of this study could be concluded: First, considering the spatial heterogeneity of the variables used in this paper, this paper extended the traditional linear regression model, and constructed a geographic weighted regression model based on spatial relations, in order to reflect the spatial correlation between PM2.5 and lung cancer mortality. Second, compared previous study using data from one city or one province, data used in this study was collected from 31 provinces ranging from 2004 to 2008 in China. Particularly, the data for dependent variable (lung cancer death) came from the “China Disease Detection System Death Monitoring Network Report Database”, which was disclosed in recent years. Third, the results of this study indicate that there is a significant positive correlation between PM2.5 concentration and lung cancer mortality. Furthermore, with the time going by, the intensity of the impact of PM2.5 on lung cancer mortality rate will increase. These findings obtained from the GWR model provide more evidence for the relationship between PM2.5 and lung cancer mortality.

Overall, this paper argues that there are rather serious environmental problems in China, and they have significant impacts on citizens’ health. The governance of air pollution should be an urgent problem for the Chinese government to solve. It is suggested that, firstly, China needs to change the economic development model at the expense of the environment. It should combine economic development with environmental protection to construct a sustainable economic development model. Secondly, the large-scale regionalization of China’s air pollution determines that China’s air governance should be coordinated. The government should take actions in the long term, but not just limited to a short-term policy in some regions. For example, during the 2008 Beijing Olympic Games, the Beijing government took strictly control measures on motor vehicles, chemical plants and other air pollution sources, but the fact showed that the effect of these measures is short-term and non-sustainable. Finally, some Chinese people, especially Chinese rural residents generally lack an effective service to deal with PM2.5 pollution. So the Chinese government should strengthen the awareness of public health protection.

For other developing countries, such as countries in South America, there are also some implications based on the present study. First, it is a dual task to develop economy and protect environment. Government of these countries should not neglect environmental protection while developing economy. Some sustainable development policies, such as raising the public awareness of environmental, and imposing a tax on polluting enterprises might be considered. Second, the universal standards which help to reduce pollution, especially air pollution, need to be formulated at the country level. Protection policies made by local government might be no-effective for the regional pollution characteristics. Third, an effective environmental pollution monitoring system and data collection system should be established over the country. Thus, government could make more reasonable measures based on information from these systems.

There are also some limitations in this study. For example, the time span of this paper was only in 2008, because the death data such as the respiratory rate of death in China is not open after 2008, so we cannot get mortality data in the longer time span. Additionally, the GWR model can only deal with cross-sectional data, and it cannot effectively deal with the panel data. However, the panel data contains a larger amount of data, so the results will be more robust. These limitations also imply the future direction for follow-up studies to measure the relationship between PM2.5 and lung cancer mortality more scientifically.

## Conclusions

In summary, this study explored the correlations between PM2.5 concentration and lung cancer mortality based on large scale data from 31 provinces, autonomous regions and municipalities in China and firstly examined the impacts of PM2.5 concentration on lung cancer mortality with the time going by. The results of the Geographic Weighted Regression Model indicated that PM2.5 concentrations is positively related to lung cancer mortality, and the relationship get stronger with time going by. These findings enhanced our understanding of the relationship between PM2.5 concentration and lung cancer mortality.

## Abbreviations

CNHFPC: China National Health and Family Planning Commission
GDP: Gross Domestic Product
GWR: Geographic Weighted Regression
IARC: International Agency for Research on Cancer
PM: Particulate Matter
WHO: World Health Organization

## Footnotes

1 Smoking has always been considered as an important factor in lung cancer. However, smoking is an individual behavior, so the impact of smoking on lung cancer is an individual micro-level. This paper aims to study the effects of macroscopic regions; therefore it did not use micro-individual data.

2 The significance test showed that PM2.5 and lung cancer mortality in 2004 and 2008 showed a significant difference at the 1% level .
